# Effect of different concentrations of pulverized mesocarp of *Citrus paradisi* Macf on the morphology and glass transition temperature of spray‐dried lemon juice powder

**DOI:** 10.1002/fsn3.678

**Published:** 2018-06-22

**Authors:** Yanilka Alcantara Marte, Gaspar Ros Berruezo, Yulisa Alcantara Marte, Andrea Escotto Tejada

**Affiliations:** ^1^ Faculty of Veterinary Sciences Department of Food Science and Nutrition Regional Campus of International Excellence “Campus Mare Nostrum” University of Murcia Espinardo Spain; ^2^ Faculty of Agrifood Science and Environment Food Technology Department Universidad ISA Santiago de Los Caballeros Dominican Republic

**Keywords:** *Citrus paradisi*, glass transition temperature, lemon powder, morphology, spray drying

## Abstract

The aim of this research was to evaluate the effect of different concentrations of pulverized mesocarp of *Citrus paradisi* Macf as a drying aid, on morphology, particle size and glass transition temperature of spray‐dried lemon juice powder. Five concentrations of grapefruit mesocarp (0.4%, 0.8%, 1.2%, 1.6% and 2.0% (w/w)) and maltodextrin DE 10 (1.2%, w/w) were evaluated as encapsulant agents. The obtained data were evaluated by one‐way ANOVA using Statistix version 8.0. For the means separation, the Tukey's test was applied with a 95% reliability. The morphology of the particles was described. According to the results, by applying different levels of coating agent in lemon juices, powder particles with different sizes (from 3.07 to 6.20 μm) and shapes (spherical, irregular and shrunken or reduced) are obtained; however, their glass transition temperature is not modified, finding values between 37.43 and 38.64°C.

## 1 INTRODUCTION

There are no coating materials with perfect encapsulation properties for the retention of volatiles compounds, protection against oxidation or with good emulsifying properties. However, the solubility, hydrophobicity, permeability and other properties of the material used as coating agent have a predominant influence on the final product′s characteristics (Lozano, 2009).

According to Arellano (2011), there is a wide variety of coating agents. Beristain (1996) indicates that each of these materials has its limitations; for instance, chemically modified starches have excellent retention of volatiles compounds during drying, but poor protection against oxidation. On the other hand, partially hydrolyzed starches (maltodextrins, glucose solids sirup and others), protect against oxidation but lack emulsifying properties. Gum arabic has excellent emulsifying properties and good retention of volatiles; however, it provides limited protection against oxidation of the encapsulated substance, and in recent years, its high cost and scarcity have limited its use.

The limited availability of materials used as encapsulating agents and the fact that they have a high cost evidences the need to identify unconventional sources of biomolecules or the like, with functional characteristics similar to those existing. This, along with the nonutilization of by‐products generated in the industrial processing of citrus fruits, led to the development of Alcantara and Escotto's (2014) study, in which an encapsulating agent was obtained from the grapefruit mesocarp.

As a follow‐up to the aforementioned study, the present investigation proposes the evaluation of different concentrations of the encapsulating agent (obtained from the grapefruit mesocarp by Alcantara & Escotto, 2014) which are evaluated in the spray drying of the Persian lemon juice. This is proposed as an alternative to the existing encapsulants in the market, so as to take advantage of a by‐product generated in the industrial processing of grapefruit, add value, and enable the industrialization and commercialization of a more durable alternative product and that conserves to the maximum the properties of the encapsulated juice.

Citrus fruits are seasonal; therefore, prices usually fall at the peak of production. For producers, this represents significant losses as the prices do not compensate the production costs. The short shelf life of these species causes losses and can negatively influence trade and consumer confidence (Alcantara & Tejada, 2012). For instance, in a given period, lemons disappear from the market or reach prices so high that most consumers cannot afford. The same happens with other tropical fruits, such as avocado, mango, pineapple, and others. An alternative to mitigate the inconveniences faced by lemon producers and consumers is processing the fruits during the high‐production season. Dehydration of Persian lemon juice was performed due to its nutritional characteristics, short shelf life, and variability in the behavior of prices, given the seasonality of its production.

Among drying technologies, spray drying is often selected for its ability to process food materials quickly and the relative control of the particle size distribution it provides (Patil, Chauhan, & Singh, 2014). Drying and storage of powdered fruit juices faces technical difficulties due to its characteristic associated with the compositions. The glass transition temperature (Tg) is one of the important properties (Hashib, Rahman, Suzihaque, Kalthum, & Ibrahim, 2015).

The aim of this research was to evaluate the effect of different concentrations of mesocarp pulverized of *Citrus paradisi* Macf as a drying aid, on morphology, particle size, and Tg of spray‐dried lemon juice powder.

## 2 MATERIALS AND METHODS

### 2.1 Raw materials

The fruits of *Citrus latifolia* Tanaka were acquired in Santiago de los Caballeros, Dominican Republic and were used to obtain the drying aid, according to Alcantara and Escotto's (2014) methodology. Maltodextrin DE 10 was used for the encapsulation of the control treatment.

### 2.2 Experimental design

For this study, a completely randomized design was used to evaluate the effect of five concentrations of encapsulant from *C. paradisi* (0.4%, 0.8%, 1.2%, 1.6%, and 2.0%), on morphology, particle size, and Tg of the encapsulated lemon juice. Additionally, 1.2% maltodextrin 10 DE was used as a control. In total, there were six treatments with three replicates, resulting in 18 experimental units.

### 2.3 Process for the lemon (*C. latifolia* Tanaka) juices encapsulation

The fruits of *C. latifolia* Tanaka were received in the Food Processing Plant of ISA University and then weighed and selected according to color, size, and appearance (without physical defects). After selection, they were treated with a sodium hypochlorite solution at 100 ppm and allowed to drain for 10 min. Then, they were weighed again and split into two halves. The juice was extracted using manual juicers and filtered using a No. 32 mesh Tyler sieve.

The treatments were prepared by adding 0.5% of tricalcium phosphate as an antiadherent (to avoid stickiness and decrease the encapsulated product's hygroscopicity), and the concentration corresponded to the encapsulating agent (*i* = 0.4%, 0.8%, 1.2%, 1.6%, and 2.0% pulverized grapefruit mesocarp; 1.2% maltodextrin DE 10). The percentages were established based on the lemon juice. It was mixed in an Osterizer 4655 electric mixer at full speed for a minute, filtered through a No. 32 Mesh Tyler sieve, to retain any possible particles and avoid obstructions in the atomization needle, and then dried in the Spray Dehydrator YC‐015 *SD*.

The drying conditions were kept constant: inlet air temperature 130°C, spray air pressure of 3.4 bar, air blower: 4 kg/cm^2^, feed rate: 0.9 L/hr, outlet air temperature 75°C. The particle size was 0.7 mm.

The obtained powder was immediately packed and vacuum sealed in bags. It was stored at 25°C until evaluation.

### 2.4 Evaluated variables

#### 2.4.1 Glass transition temperature (Tg)

This measurement was made using a DSC 822e Mettler‐Toledo (Barcelona, Spain), equipped with cooling accessories with liquid nitrogen. 10 mg of encapsulated lemon juice powder was weighed in a standard 40 μl aluminum melting pot (ME‐51119870; Mettler‐Toledo), closed with a standard aluminum cap (ME‐51119871; Mettler‐Toledo) and hermetically sealed. The analysis was carried out directly without thermal history erasure, with a sweep from −20 to 120°C, with a heating rate of 10°C/min. The midpoint of the glass transition was considered as the characteristic temperature of the transition.

#### 2.4.2 Particles size

For this determination, samples were fixed to the sample holder with double‐sided carbon tape (Ted Pella Inc.) and metallized with platinum in a Polaron coater (model SC7640 Quorum Technologies) for 120 s at 15 mA. A scanning electron microscope (SEM) Hitachi S‐3500N (Hitachi High‐Technologies Corporation, Tokyo, Japan) at 15 kV acceleration voltage was used to observed samples. A working distance between 7 and 8 mm was applied and under high vacuum conditions. The signal with which the image was generated was secondary electrons (SE Secondary Electron). For the acquisition of the images, the Esprit Quantax 400 program (Bruker Nano GmbH, Berlin, Germany) was used and the Quartz PCI version 5.1 of the Quartz Imaging Corporation were used, both for acquisition and for taking measurements.

#### 2.4.3 Morphology of particles

To analyze the morphological characteristics of the microcapsules and the possible aggregation of the particles of dried Persian lemon juice by spray, the scanning electron microscopy (SEM) technique was applied, using the JEOL JSM 5900LV equipment, according to the methodology described by Lozano (2009).

The powder samples obtained from each treatment were fixed to a double‐sided adhesive tape placed in a sample holder or canyon. A small sample spatula tip was applied, fixing it to the tape by means of a small pressure. Later, they were metallized with gold for 240 s, using an SPI Module for cathodic sputter coating. This was performed with the aim that the small dust grains were more stable and did not move when the microscope lens was near them to visualize them, increase the conductivity of the samples and obtain images with good resolution. The lens was placed 7 mm away. All the images were taken looking for a representative field of the sample that was as flat as possible, operating at 5 kV and using a magnification of 1,000×.

### 2.5 Statistical analysis

The obtained data were evaluated by one‐way ANOVA. Means were separated using Tukey's test (*p < *0.05). These analyses were performed using Statistix version 8.0. For the representation of the results, the arithmetic mean was used as the central measure ± *SD* of three replicates.

## 3 RESULTS AND DISCUSSION

### 3.1 The effect of different concentrations of pulverized mesocarp of *C. paradisi* Macf on Tg of spray‐dried lemon juice

In Table 1, it is observed that the amount of encapsulant used in juices had no effect on the Tg. Probably because the differences, in quantities of drying aids added, were minimal. Similarly, Fang and Bhandari (2012) found no effect on Tg of encapsulated bayberry juice powders using 0.5%, 1.0%, 2.5%, 5.0%, 7.5% and 10% of whey protein isolate.

**Table 1:** Vitreous transition temperatures and particle diameters of encapsulated lemon juice powders

Encapsulant concentration (%)	Start of vitreous transition (Onset) (°C)	Midpoint of glass transition temperature (Tg) (°C)	Particles′ size (μm)
0.4	31.10^a^ ± 0.83	37.56^a^ ± 0.47	5.51 ± 0.54^b^
0.8	31.02^a^ ± 2.26	38.11^a^ ± 2.04	6.20 ± 0.50^a^
1.2	31.06^a^ ± 1.71	37.55^a^ ± 0.29	3.07 ± 0.52^c^
1.6	31.81^a^ ± 3.68	38.47^a^ ± 2.41	6.08 ± 0.72^ab^
2.0	31.03^a^ ± 0.68	38.64^a^ ± 0.55	5.61 ± 0.44^ab^
1.2 Maltodextrin	30.38^a^ ± 4.23	37.43^a^ ± 3.70	5.75 ± 0.17^ab^

Different letters between rows indicate significant statistical differences (*p* < 0.05) between the means of the treatments evaluated. The data analyzed are expressed as means ± *SD* (*n* = 3 for glass transition and *n* = 10 for particle diameters). MD: maltodextrin 10 DE.

This study's results are different from those found by Hashib et al. (2015), where the concentration of drying aids (15%, 20% and 25% maltodextrin DE‐5) had effects on Tg of pineapple powder. Higher relationship between maltodextrin and pineapple juice conduce to raise the Tg of encapsulated product (an increase in maltodextrin concentration from 15% to 20% raised the Tg of pineapple powder from 149.24 at 152.90°C). These authors explain that maltodextrin's effect on the increase in the Tg of juice is due to its high molecular weight, which increases the juice temperature (Jittanit, Niti‐Att, & Techanuntachaikul, 2010; Tonon, Brabet, Pallet, Brat, & Hubinger, 2009). Additionally, Goula and Adamopoulos (2010), dried orange juice by spray, using concentrations of maltodextrin DE‐6 of 2.5%, 5%, 10% and 40%, obtaining an increase in the Tg of 33, 53, 78 and 123°C, respectively. Hashib et al. (2015) clarify that it should be taken into account that DE‐6 has a lower molecular weight compared to DE‐5, resulting in a lower Tg.

**Figure 1:**
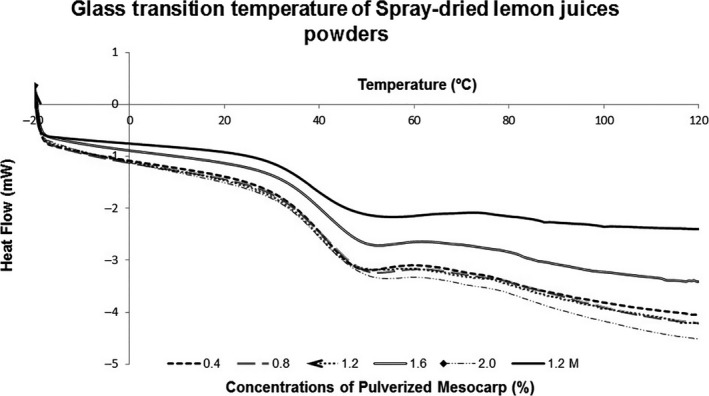
Vitreous transition temperature of atomized lemon juice powders using different percentages of grapefruit mesocarp as encapsulant and the control (with maltodextrin).

In the present research, both the start of vitreous transition (Onset) and the midpoint of Tg (Midpoint) were lower for the juice encapsulated in maltodextrin DE‐10. Figure 1 displays that all treatments showed the same behavior.

According to Bhandari, Datta, Crooks, Howes, and Rigby (1997), Tg of glucose is 31°C. In relation to this study's results, the main sugar contained in the encapsulations is glucose, as it is freshly squeezed juice, without the addition of sweeteners or sugars. Lozano (2009) explains that lower values of Tg usually correspond to saccharides of low degree of polymerization. According to Mani, Jaya, and Das (2002), the presence of sugar (sucrose, fructose and glucose) in fruit juice results in a low Tg, the temperature at which an amorphous solid begins to change from vitreous state to a gummy state. In this way, the tackiness of powders can be related to their Tg. In order to reduce stickiness, the Tg of the powdered juice can be manipulated by the addition of carrier agents such as maltodextrin, a high‐molecular‐weight powdery substance (Chegini & Ghobadian, 2007).

Fang and Bhandari (2012) stated that Tg of spray‐dried powders is a very important indicator to assess whether a drop/particle is likely to stick to the spray dryer wall. The practical rule of Bhandari et al. (1997) indicates that generally stickiness occurs if the temperature of the drop/particle is 20°C above its Tg. According to the rule's analysis, when adding the 20°C to the Tg of encapsulated lemon juice powders (37.43–38.64°C), its sticky temperature should be higher than 57.43 to 58.64°C. These values are higher than a particle's typical surface temperature during drying by spray (40–50°C, according to Masters, 1991). This fact indicates that the recovery of dust must be high. Fang and Bhandari (2012) managed to reach those values by adding amounts greater than or equal to 30% maltodextrin DE‐10 and reported a dust recovery of more than 50%; however, this study's Tg values are higher than those found by Fang and Bhandari (2012), in powders of bayberry juice encapsulated in 0.5%–10% of whey protein isolate (14.12–15.34°C) and similar to those found by the same authors in bayberry juice powders encapsulated in 50% maltodextrin DE‐10 (37–38°C).

### 3.2 The effect of different concentrations of encapsulant retrieved from *C. paradisi* Macf mesocarp on particles’ size of spray‐dried lemon juice

Table 1 shows that the amount of encapsulating agent used in the lemon juice influenced the particles’ diameter of the resulting powders; however, there was no linear behavior. This could be because of the size of particles undergoing changes during spray drying, related to moisture content (Ray, Raychaudhuri, & Chakraborty, 2016) and the high concentration of solids in the solution (Bimbenet, Bonazzi, & Dumoulin, 2002).

This study's results are different from those found by Hashib et al. (2015), where the particle size of pineapple dusts decreased with the increase in encapsulant content (15%, 20% and 25% maltodextrin DE‐5).

Villacrez (2013) found that the particle size depends directly on the encapsulating agent and the way it interacts with the active material and not of the internal diameter of the spray nozzle used for the drying process.

In Table 1, it can be seen that the range obtained for the diameter of the particles of the lemon juice powders was 3.07 ± 0.52 to 6.20 ± 0.50 μm, being within the values considered for spray‐dried products: 3–100 μm, according to Ray et al. (2016) and less than 40 μm, according to Zuidam and Heinrich (2010).

The values found for the particles of atomized lemon juice are higher than those reported by García‐Cárdenas, Ciro‐Velásquez, and Largo‐Ávila (2015), in citrus flavor of encapsulated tangerine using 32% of a mixture of different covering materials (10 DE maltodextrin, Arabic gum and soy protein), ranging between 0.6 and 2.7 μm.

Only the powder's size obtained with 1.2% grapefruit mesocarp as drying aid resembles the results reported by Paramita, Furuta, and Yoshiia (2010), for limonene encapsulated by combinations of Arabic gum, maltodextrin and an emulsifying agent (1.41–3.43 μm) and Soottitantawat et al. (2005), for encapsulated d‐limonene using a mixture of Arabic gum and maltodextrin (0.84 to 3.37 μm).

Ordoñez and Herrera (2014) reported particle sizes from 4 to 17 μm for matrices encapsulated in Arabic gum with cassava starch and limonene 50:50 and from 1.5 to 14 μm for matrices encapsulated in Arabic gum with cassava starch and limoneno 17:83. Additionally, they obtained particle sizes from 2 to 13 μm for matrices encapsulated in whey protein concentrate and whey protein concentrate with cassava starch and limonene and from 1 to 9 μm for Arabic gum with limonene.

### 3.3 Morphology of spray‐dried lemon juice

**Figure 2:**
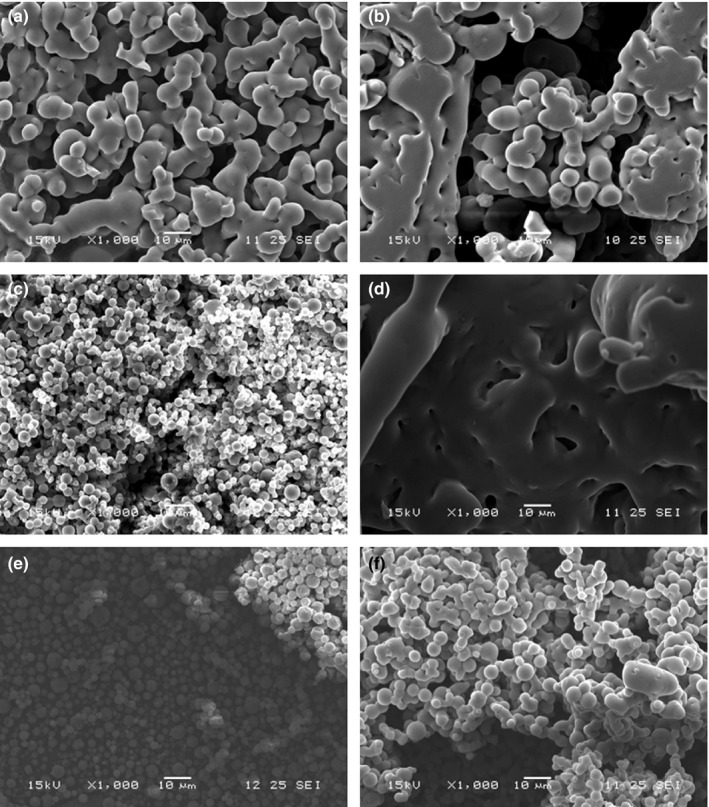
SEM photographs of the atomized lemon juice samples with different percentages of Mesocarp Powder Encapsulating Agent of Grapefruit: (a) 0.4; (b) 0.8; (c) 1.2; (d) 1.6; (e) 2.0; (f) 1.2% Maltodextrin DE‐10. Magnification 1,000×

Figure 2 presents the images of lemon juice samples atomized using different percentages of pulverized encapsulating agent of mesocarp of grapefruit and the control of maltodextrin DE‐10, taken with an Electronic Scanning Microscope. It can be seen that the particles of the juices obtained at different levels of the encapsulating agent have different sizes and shapes (spherical, irregular and shrunken or reduced), coinciding with that observed by Bhusari, Muzaffar, and Kumar (2014) in tamarind pulp in spray‐dried powder using 40%, 50% and 60% maltodextrin DE‐20 and Arabic gum.

Atomized juices with 1.2% and 2.0% of mesocarp encapsulant and 1.2% of maltodextrin had a better defined spherical shape. In these dust particles, a continuous wall is observed with no cracks in the surface. These findings were also reported by Bhusari et al. (2014) in spray‐dried tamarind pulp using 40%, 50% and 60% maltodextrin DE‐20.

García‐Cárdenas et al. (2015) also report having found almost complete spheroids without practical evidence of broken microcapsules in a tangerine citrus flavor encapsulated in different covering materials. They explain that this characteristic is indicative of the microcapsules’ good structure found in the final product (powder).

The other percentages of encapsulant studied have very varied forms, being those of 1.6% the least similar to the others and not presenting fractions of spherical particles (they form sticky aggregates). The morphology exhibited by the atomized juice with 0.4% and 0.8% grapefruit encapsulant is similar to that observed by Lozano (2009), in *Opuntia stricta* juice samples sprayed with a ratio 1.8 of fructooligosaccharide Beneo P95/juice; while the particles of the atomized juice with 1.6% of grapefruit mesocarp are more similar to that observed by the same author using ratios of fructooligosaccharide Beneo P95/juice of 0.45 and 0.90.

## 4 CONCLUSION

The amount of encapsulant used in *Citrus latifolia* Tanaka juice did not change the glass transition temperature of the powders obtained by spray drying.

By applying different levels of coating agent in lemon juices, powder particles with different sizes and shapes (spherical, irregular and shrunken or reduced) are obtained.

From the doses used in this research, it was determined that 1.2% of grapefruit mesocarp could be used as an encapsulant for lemon juice during spray drying.

## ETHICAL STATEMENT

This study does not involve any human or animal testing.

## CONFLICT OF INTEREST

None declared.
